# Assessing the potential of bioeconomy in Slovakia based on public perception of renewable materials in contrast to non-renewable materials

**DOI:** 10.1007/s13280-020-01368-y

**Published:** 2020-09-11

**Authors:** Lenka Navrátilová, Jozef Výbošťok, Zuzana Dobšinská, Jaroslav Šálka, Magdaléna Pichlerová, Viliam Pichler

**Affiliations:** 1 Department of Forest Economics and Management, Faculty of Forestry, Technical University in Zvolen, T. G. Masaryka 24, 960 01 Zvolen, Slovakia; 2 Department of Planning and Landscape Design, Faculty of Ecology and Environmental Sciences, Technical University in Zvolen, T. G. Masaryka 24, 960 01 Zvolen, Slovakia; 3 Department of Natural Environment, Faculty of Forestry, Technical University in Zvolen, T. G. Masaryka 24, 960 01 Zvolen, Slovakia

**Keywords:** Bioeconomy, Bioeconomy development, Perception of materials, Renewable natural resources

## Abstract

There is a need for societal transformation towards bioeconomy, which promotes the replacement of non-renewable natural resources with renewable ones. Slovakia has considerable potential for bioeconomy development, yet this potential remains untapped. This article evaluates the public perception regarding the individual properties of renewable and non-renewable materials and their relation to the potential for bioeconomy development in Slovakia. It is found that Slovak consumers prefer natural renewable materials, regardless of other influencing factors, and realise the need for transformation towards a more sustainable economy.

## Introduction

At present, the utilisation of natural renewable resources is receiving increasing attention, both globally (EC 2018; UNEP 2011) and locally (Kleinschmit et al. 2017; Winkel 2017). Owing to their impact on life quality, health, and ecosystems (Mac Kinnon et al. 2018), efforts are being made to replace non-renewable natural resources in energy production (EC 2005; Yang et al. 2016), manufacturing (Chen and Chai 2010), and services (Haldar 2019; Litardi et al. 2020), with renewable alternatives (Donia et al. 2018).

The concept of bioeconomy, also known earlier as knowledge-based bioeconomy, was launched in 2005 at an international conference held by the European Commission, and the future perspectives of bioeconomy were defined in 2007 (Birch et al. 2012; EC 2005). In 2012, the European Commission established a strategy and action plan for bioeconomy, in a document titled: “Innovating for sustainable growth: A bioeconomy for Europe”. This document describes bioeconomy as an economy that “encompasses the production of renewable biological resources and the conversion of these resources and waste streams into value added products, such as food, feed, bio-based products and bioenergy” (EC 2012). Using this document as a reference, European countries began to make efforts to publish their own bioeconomy strategies (Biookonomierat 2015). In 2018, the European Commission published an updated bioeconomy strategy: “A sustainable bioeconomy for Europe: strengthening the connection between economy, society and the environment”. The document formulates a set of 14 specific actions designed to tackle global societal challenges (EC 2018).

The understanding and definitions of the concept of bioeconomy differ among countries, and so do the pathways chosen to promote the bioeconomy strategies according to prerequisites of the individual countries (Staffas et al. 2013). Bioeconomy in Slovakia is not governed by an explicit bioeconomy strategy, although several bioeconomy-related strategies can be found in different sectors, such as forestry, agriculture, energy, environment, and rural development.

The use of renewable natural resources in the production of food, energy, materials, and other products is the core aim of the bioeconomy strategy (Schmid et al. 2012). The main characteristic of renewable natural resources is that appropriate management of these resources can ensure their availability for future generations. In contrast, once consumed, non-renewable natural resources cannot be recovered and reused in the near future. This can cause serious problems, such as resource scarcity and economic crisis (Tietenberg 2015). Wood is one of the main natural renewable resource. Nowadays, it has a wide range of uses, including the production of paper, furniture, wooden constructions, energy, the modern production of textiles, and chemicals. In Slovakia, the share of renewable energy resources was only 8.2% in 2019. Oil, coal, gas, and nuclear power prevail (Ministry of the Environment 2020). Innovative products are being adopted reluctantly, as most people prefer known products and materials. Slovakia belongs to the moderate innovators group according to the 2019 European innovation scoreboard. This results in an advantage (creates an opportunity) for natural materials, such as wood, that is perceived as a traditional material. This is also supported by previous research (Kaputa et al. 2018; Paluš et al. 2012), which has highlighted that wood and wood-based products are perceived positively by Slovak consumers.

Oil-based materials are also well known and widely used, yet it is not desirable that the use of these materials continues in the future. Over the past 10 years, there was a mild decline in the use of oil-based materials in Slovakia, caused by their decreasing popularity and, most probably, by increased energy efficiency (Ministry of the Environment 2020). In 2017, 13.8 million tonnes of oil-based materials were consumed, while in 2009 the consumption was of 14.8 million tonnes. The consumption of metal has slightly increased over the past 10 years. In 2017, 3.5 million tonnes of metal were consumed compared to 2.3 million tonnes in 2009 (Eurostat 2019c). The consumption of biomass in 2015 was nearly four times higher than that in 1990 (Ministry of the Environment 2020). Current forecasts indicate that biomass consumption will increase by 12% by 2030, and that this consumption will mostly be for energy generation purposes (Ministry of the Environment 2020).

This forecast (probably) also accounts for the growing array of bio-based materials (often also referred to as biomaterials), which are a new generation of materials derived from biomass (living matter, such as plants, trees, algae, marine organisms, microorganisms, and animals) that have undergone extensive processing, such as viscose or rayon (Curran 2010).

The use of renewable materials is expected to help reducing the greenhouse gas (GHG) emissions as they replace non-renewable resources and materials because the GHG emissions related to renewable materials are already accounted for in the carbon budget (Lewandowski et al. 2018). Bioeconomy is therefore focused on supporting economic growth and simultaneously reducing GHG emissions. According to recent studies, to keep the global warming below 1.5 °C, half of the gas reserves, a third of the oil reserves, and over 80% of the coal reserves should not be used in the period 2010–2050 (McGlade and Ekins 2015).

So far, there has been a reduction of GHG emissions in the EU by 22.4% compared to 1990 levels. Therefore, there is an expectation to exceed the European target of GHG emissions reduction by 2020^1^ (Eurostat 2019a, b). The current share of renewable energy sources in final energy consumption in Slovakia (including electricity, heating and cooling, and transport) is 17%, while the rate of renewable sources in our electricity consumption is 29.6%^2^ (Eurostat).

Materials used in manufacturing have a great impact on economic sustainability and success; this is why attention is given to bio-based materials, which are being developed worldwide. The focus of researchers is on biomass (Scarlat et al. 2015), bio-based plastics (Philp and Krishna 2013), biofuels (Philp 2015), and biorefineries (Stafford et al. 2020). Several studies also focus on perception, especially of bio-based products (Bracco et al. 2019). This attention is understandable, as there is an ongoing change in the demand of materials, influenced by concepts such as sustainability and bioeconomy (Laibach et al. 2019; Lynch et al. 2016). On the other hand, little focus is given on consumers’ preferences and public perception of different materials (traditional and biomaterials) in relation to bioeconomy. Various studies have focused on the perception of specific materials by the general public, such as plastic (Dilkes-Hoffman et al. 2019b), bioplastic (Dilkes-Hoffman et al. 2019a; Lynch et al. 2016), bio-based products (Lynch et al. 2016; Sijtsema et al. 2016), wood and wood products (Kitek Kuzman et al. 2012), but also by stakeholders, for instance of biomass (Dwivedi and Alavalapati 2009), and of bioeconomy in general (Imbert et al. 2019).

In the case of Slovakia, wood biomass is recognised as the most important renewable energy source in terms of usability. As aforementioned, we have witnessed an increase in biomass utilisation in Slovakia. Its full potential, however, remains untapped (EC 2018). Several studies have focused on environmental attributes of materials (mostly wood and wood products) (Kaputa 2006; Kaputa et al. 2018; Paluš et al. 2012), as well as on environmental awareness in Slovakia (Miklenčičová 2015). According to Paluš et al. (2012), even though consumer preferences are permanently changing due to innovations and changes in lifestyle, the ecological properties of wood and wooden products remain important when it comes to decision making. As stated by Kaputa, the majority of wood processing companies in 2006 did not find the consumers to be environmentally conscious. Nowadays, this is not the case anymore. According to Kaputa et al. (2018), “every person experiences the world in a different way, and reality for the individual is only what is perceived to exist or what occurs”. Within this logic, many consumers in Slovakia today see the need to minimise the negative impact of materials and products on the environment (Paluš et al. 2012).

To date, previous studies have examined public perceptions of individual materials or groups of renewable materials. This study aims to evaluate differences in the perception of non-renewable and renewable materials and draw conclusions about whether and how they can be used towards promoting or developing bioeconomy. Our main research questions can be stated as follows: (1) how does the Slovak public perceive various kinds of renewable and non-renewable materials?; (2) in view of the public perception of different materials, does bioeconomy, as a new policy, have the potential to develop in Slovakia?

The objective of this study is to identify the potential for bioeconomy development in Slovakia in accordance with the public perception of several kinds of materials (bio-based materials, plastic, other oil-based materials, wood, other natural materials, paper, glass, and metal). The results could facilitate the development of national bioeconomy strategies in countries where they are absent (such as in Slovakia), or could help refine these strategies where they have already been deployed. In fact, bioeconomy strategy must inevitably deal with consumers’ preferences in materials, which reflect their environmental awareness and perceived need for transformation towards the bioeconomy.

## Materials and Methods

In order to analyse the potential for bioeconomy implementation in Slovakia, a survey was undertaken to identify consumer preferences towards different kinds of materials, both renewable and non-renewable ones.

### Sample size

The intention was to carry out the survey on a representative sample of respondents. The population size for each stratum was known; therefore, the sample size for each stratum was determined using formula [1] according to Krejcie and Morgan (1970):

1 $$n = \frac{{x^{2} NP\left( {1 - P} \right)}}{{d^{2} \left( {N - 1} \right) + x^{2} p\left( {1 - P} \right)}}$$

where *n* is the required sample size, *x* is the table value of Chi-square for 1 degree of freedom at the expected confidence level (1.645), *N* is the population size, *P* is the population proportion (0.5), and *d* is the degree of accuracy expressed as a proportion (0.10).

The required sample size and the real sample size are shown in Table 1. The real sample size reaches in most cases the required sample size, except for the group of respondents over 61 years old, for which the margin of error is 14.32% instead of the required 5%.

**Table 1:** Determination of sample size

Variable	Stratum	Population size	Required sample size (error 5%, CL 90%)	Real sample size (*n*) *N* = 538	Real margin of error (CL 90%)
Gender	Men	2 661 077	271	272	4.99
	Women	2 789 344	271	266	5.04
Age	18–35	1 102 658	271	224	5.49
	36–60	1 985 239	271	281	4.91
	61>	1 239 705	271	33	14.32
Residence	Urban	2 912 062	271	319	4.60
	Rural	2 538 359	271	219	5.56

^a^http://statdat.statistics.sk

### Data collection

The survey was originally conducted by Kairos Future, an international consulting and research company. In January 2016, the survey was administered in the United States, Brazil, China, Sweden, and Germany. The survey was administered in Slovakia and Italy in February 2018, by the Forestry Faculty of the Technical University in Zvolen, Slovakia, within the framework of the H2020-MSCA-RISE-2016-CHARMED project.

We used the purposive sampling technique to select the respondents. The survey was distributed via email to the digitally literate population segment, thus representing the digitally conscious, educated, urban segment of the population best, i.e. the global middle-class consumer of the future. A standardised method was used to collect qualitative data through the survey, including the basic information about the respondents, such as gender, year of birth, education, and employment. Overall, the survey consisted of 68 main questions focusing on the environment, of which 60 were closed-ended and 8 were open-ended questions. In this study, we only used data from Slovakia. We focused on questions about renewable and non-renewable materials (wood; other natural materials, namely wool, cotton; paper; bio-based materials, namely viscose and rayon; glass; metal; plastic; other oil-based materials, namely polyester and nylon) and 12 of their attributes (modernity, lastingness, naturality, excitement, quality, exclusivity, beauty, traditionality, advance, timelessness, eco-friendliness, and reliability). We examined to what extent our respondents associated the attributes to the materials on a 1–7 scale, with 1 indicating “strongly no association”, 2 indicating “no association”, 3 indicating “less association”, 4 indicating “undecided”, 5 indicating “more association”, 6 indicating “association”, and 7 indicating “very strong association”.

The public perception could be affected by several factors. In this work, we focus on three factors: age, gender, and residence. Based on age, we focused on examining the effect of age on the perception of different materials. Respondents consisted of three groups: young—18–35 years old, middle-aged—36–60 years old, and old—61 years old and older. Based on gender, the sample consisted of two categories (women and men). As for residence, we focused on examining whether the respondents lived in urban or rural areas. In the urban population, we included respondents who have identified their place of residence as ‘urban’ or ‘predominantly urban’, whereas in the rural population we included respondents who have identified their place of residence as ‘rural’ or ‘mostly rural’.

### Statistical analysis

To analyse the data, we used the open-source statistical program R. The properties of each material were analysed simultaneously, and the individual attributes of each material were also evaluated. In doing so, we first determined the median of the most common response for each material, followed by the average of responses for the material. In the subsequent step, we tested the normality of the data distribution by the Kolmogorov–Smirnov test (Table 2).

**Table 2:** One-sample Kolmogorov–Smirnov (*Z*) test

Material	*N*	Mean	Std. deviation	Kolmogorov–Smirnov (Z)	Sig. (2-tailed)
Bio-based	5775	3.92	1.59	13.68	0.000
Glass	5564	4.71	1.65	10.98	0.000
Metal	5510	4.3	1.69	11.39	0.000
Other natural	5625	4.85	1.62	11.11	0.000
Other oil-based	5637	3.13	1.64	11.11	0.000
Paper	5563	4.26	1.71	11.79	0.000
Plastic	5685	3.15	1.7	11.09	0.000
Wood	5530	5.21	1.69	14.99	0.000

The test showed that the data distributions are significantly different from normal distributions. Therefore, we analysed the influence of gender, residence, and age through the Chi-squared test.

### Findings

**Fig. 1:**
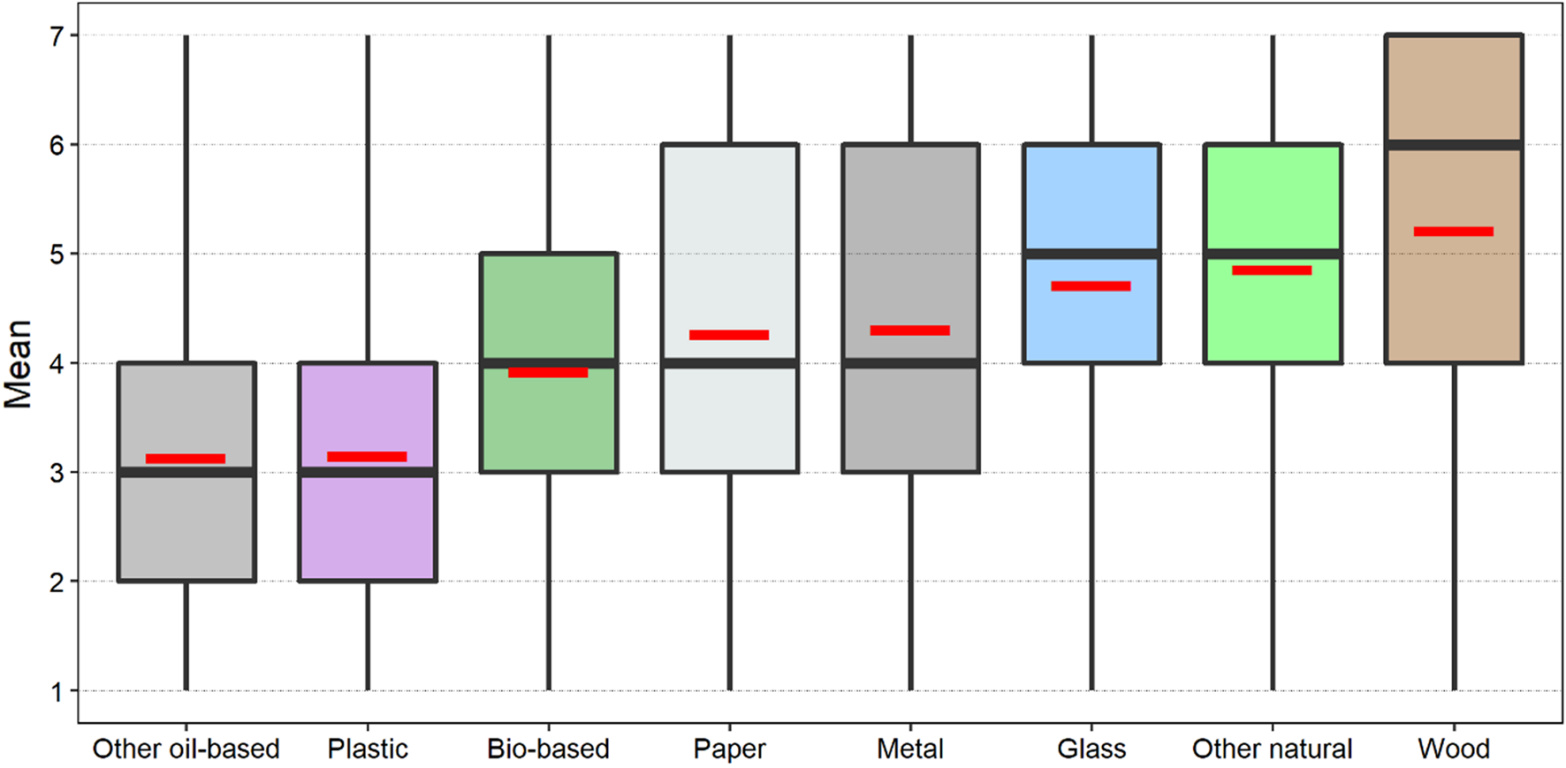
Boxplot of individual materials as perceived by respondents (black thick line represents median of responses, red line symbol represents average of responses, box represents 25th and 75th percentile, whiskers represent the variability outside the upper and lower quartiles)

The results of our analyses (Fig. 1) highlight how different materials are perceived by the Slovak public in general. At the top of the list are wood, other natural materials (e.g. cotton, wool) and glass, followed by metal, paper, and bio-based materials. At the bottom of the list are plastic and other oil-based materials (e.g. polyester, nylon). Respondents perceive wood as the best option: the average value of the responses is about 5.2, with a median value of 6. The box representing wood is also relatively tall, which indicates a great variability of responses, predominantly in the less positive quartile group, as shown by the long lower whisker.

Natural materials and glass have a very similar distribution, exhibiting a median value around 5. The boxes representing these two materials are relatively short, indicating a lower level of response variability. The considerably longer lower whisker indicates that differences in opinions are to be found in the less positive quartile group.

As for metal, paper, and bio-based materials, the median value of the responses was 4. The distributions of responses for metal and paper are almost identical; both boxes are relatively tall with longer upper whiskers, indicating large response variability, predominantly in the more positive quartile group.

On the other hand, the box representing the bio-based materials is relatively short, with equal upper and lower whiskers indicating a small variability in the responses, with differences in opinions being evenly distributed in the less positive and more positive quartile groups.

According to the results, the perception of oil-based materials is beginning to worsen. The median value of responses for plastic and oil-based materials is 3; the average response values are almost identical, around 3.1. The boxes representing both materials are relatively short, with considerably longer upper whiskers indicating relatively small variability of responses, with differences being predominantly in the more positive quartile group.

**Fig. 2:**
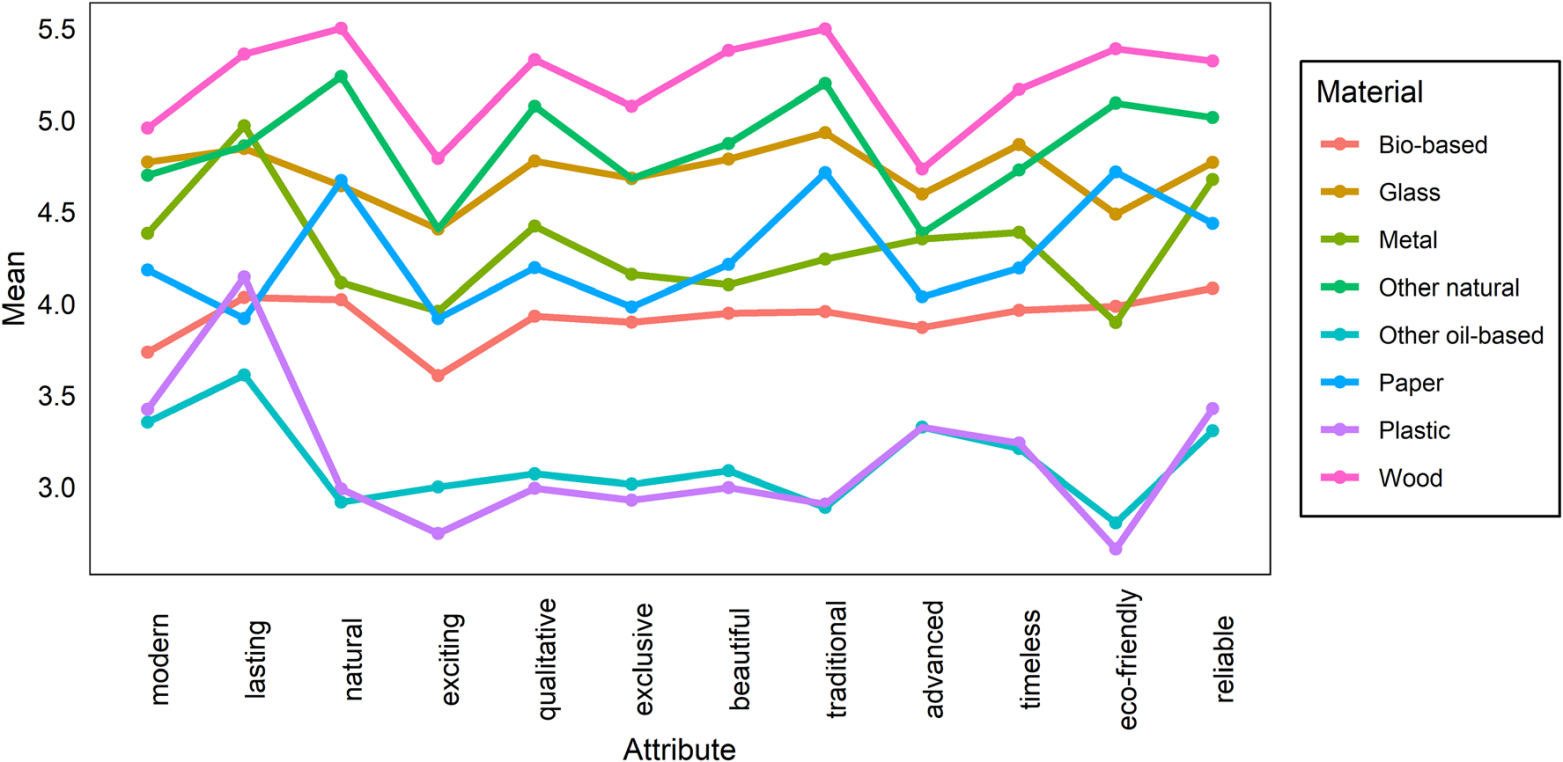
Mean value of perception of particular attributes for each material

Subsequent analyses were focused on the attributes of each material and on the way they are perceived by the Slovak public. Figure 2 shows the differences in perception of different attributes for each material. In the case of wood, the highest ranked attributes were naturality and traditionality, closely followed by eco-friendliness, lastingness, quality, beauty, and reliability. The lowest ranked attributes were timelessness, exclusivity, modernity, excitement, and advance. Nonetheless, wood consistently ranked first in each attribute. Other natural materials, such as cotton or wool, showed very similar trends, with similar highest and lowest ranked attributes.

Regarding glass, the top ranked attributes were traditionality and timelessness (similar to the previously discussed materials), but also lastingness. The lowest ranked attributes were eco-friendliness and excitement. In the case of paper, the analyses show the top ranked attributes to be eco-friendliness, traditionality, and naturality. This result is very similar to that for wood and other natural materials. Similarities can also be found in the lowest ranked attributes, which for paper are exclusivity, lastingness, and excitement. The ranking of attributes for the bio-based materials are almost identical, with only slight deviations. The best ranked attributes for the bio-based materials are reliability, lastingness, and naturality; the worst ranked are modernity and excitement.

Finally, plastic and other oil-based materials also show similar attribute rankings, with the same top and bottom ranked attributes. For both materials, lastingness, reliability, and modernity rank at the top, followed by timelessness and advance. At the bottom, we find excitement, traditionality, and eco-friendliness.

**Fig. 3:**
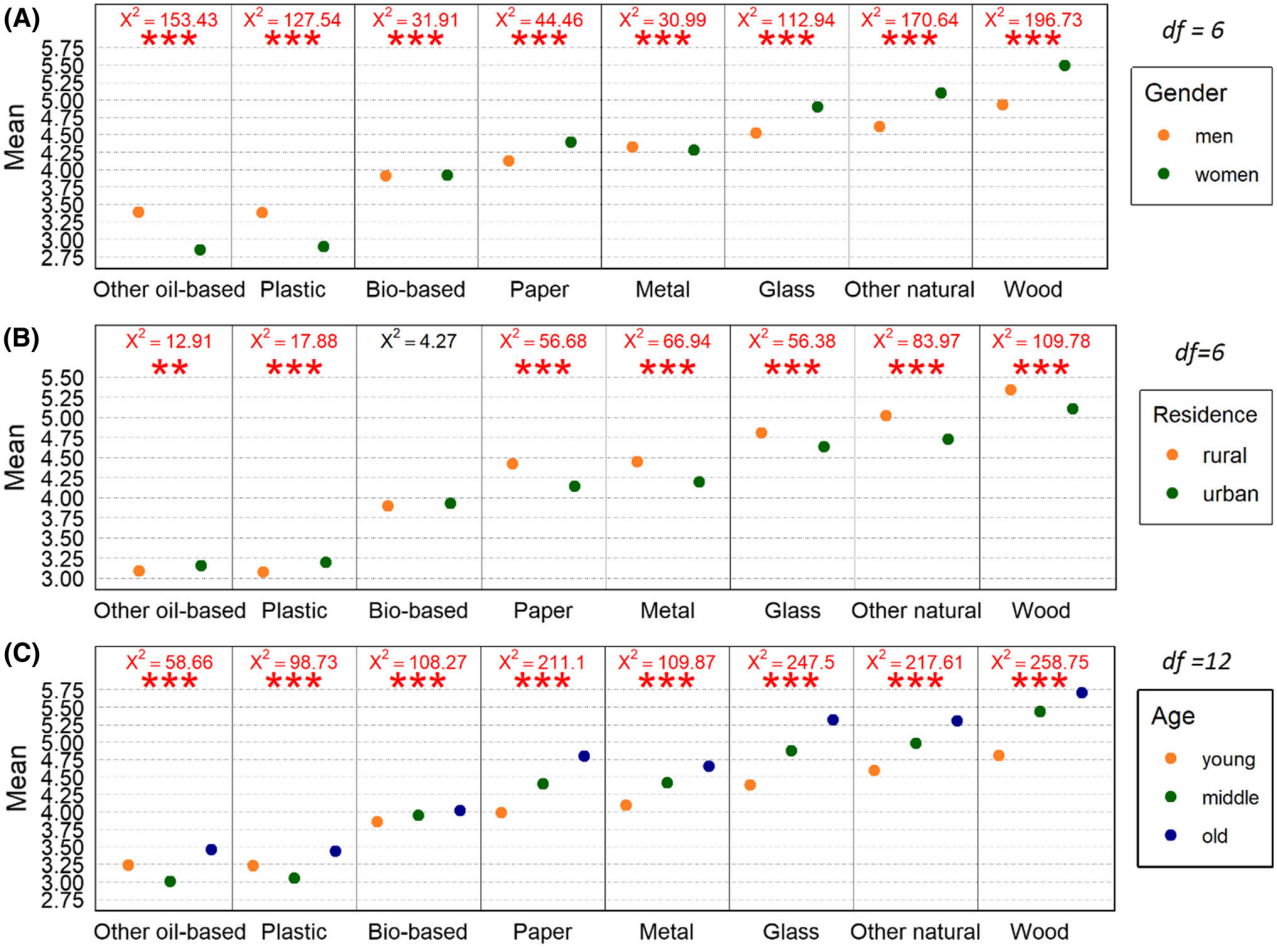
Average and Chi-squared value of response on material perception by men and women (**a**), rural and urban residents (**b**) and young, middle-aged and older respondents (**c**) (*p<0.05, **p < 0.01, ***p < 0.001)

Furthermore, we examined if and how gender influences the perception of these materials (Fig. 3a). In general, the Chi-squared test showed significant differences in the perception of all materials between men and women. Women assign significantly higher values to wood, other natural materials, glass, and paper in comparison to men. At the bottom of the list are plastic and oil-based materials, to which women assign significantly lower values compared to men. It can be concluded that both groups of respondents prefer natural and more “eco-friendly” materials over oil-based materials, but this preference is significantly stronger in women.

Subsequent analyses were focused on determining the influence of the place of residence on the perception of bioeconomy (Fig. 3b). The results show significant deviations in the perception of most materials, with the exception of bio-based materials. Rural residents have a much more positive perception of wood, other natural materials, metal, and paper. Glass is perceived slightly more positively by rural residents than by urban residents. On the contrary, plastic is perceived slightly more positively by urban residents. Concerning bio-based materials and other oil-based materials, no deviation was found, as both groups of respondents favour natural materials over oil-based materials. For rural respondents, this preference is understandably stronger, as rural residents are more connected to nature compared to urban residents.

Next, we examined how the age of respondents influences the perception of the materials (Fig. 3c). We identified deviations in the perception of all materials. Old respondents perceive all materials more positively than young and middle-aged respondents. Their perception of wood, other natural materials, glass, metal, and paper is considerably higher compared to young respondents, and slightly higher compared to middle-aged respondents. The largest difference in the perception of plastic is found between old and middle-aged respondents. This holds true also for the perception of oil-based materials.

**Fig. 4:**
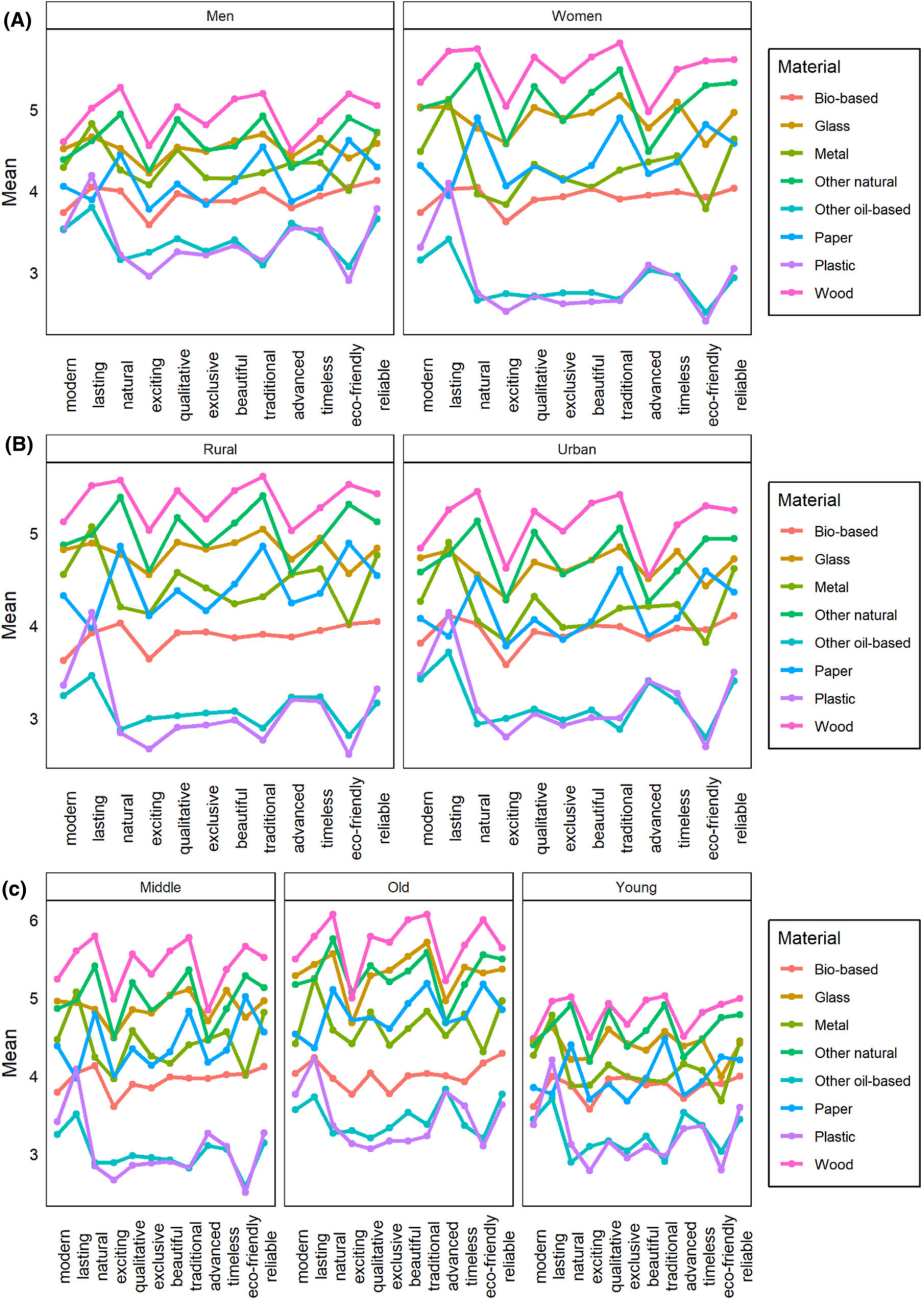
Perception of individual attributes for each material by women and men (**a**), rural and urban residents (**b**) and young, middle-aged and older respondents (**c**)

In addition, we analysed the perception of attributes for each material based on gender (Fig. 4a). The results show a higher variance in the responses given by women than in those given by men. Men tend to assign lower values to all attributes for almost each material, with the exception of plastic and other oil-based materials. Women find wood more lasting, traditional, and reliable than men. They also find other natural materials more beautiful, timeless, and reliable. In the case of glass, the trend is the same for men and women; however, the values assigned for each of the attributes by men are lower than those assigned by women. On the contrary, men perceive metal to be markedly more natural, exciting, eco-friendly, and having higher quality.

The analyses for paper show the same results as for glass: the trend of responses is the same for men and women; however, the values assigned for each attribute by men are considerably lower than those assigned by women. The results for bio-based materials show that men perceive them as being of higher quality, more traditional, and more eco-friendly compared to women. Men also perceive bio-based materials as less beautiful and advanced. The trend of responses for plastic and other oil-based materials is, again, very similar for men and women, yet with lower values assigned by women than by men. With regard to plastic, women perceive it considerably less natural, exciting, and eco-friendly than men. Regarding other oil-based materials, the only differences (besides the lower values assigned by women) are in the perception of their quality and beauty, which is considerably higher for men than for women.

In the next step, we analysed the perception of the attributes of each material by urban and rural residents. Figure 4b shows the results of this analysis. Regarding wood and its attributes, we can see the same trend of responses by both rural and urban residents, with slightly lower values assigned by the latter compared to the former. Rural residents find wood to be more lasting and traditional than urban residents. Urban residents also assign markedly lower values to the “advance” attribute than rural residence, for whom it reaches the same score as glass.

In the case of other natural materials and glass, we can see the trend of responses being the same for rural and urban residents, with lower values assigned by urban residents. Regarding other natural resources, rural residents assign higher values to beauty and eco-friendliness.

Concerning metal, the figure shows markedly lower values being assigned to the excitement, advance, and timelessness attributes by urban residents compared to rural residents. Paper and bio-based materials show the same trend for rural and urban residents, with lower values assigned to all attributes by urban residents. On the contrary, rural residents perceive paper to be more natural and beautiful than urban residents. Plastic and other oil-based materials also show the same trend of responses by rural and urban residences, with only slight deviations. Rural residents perceive plastic to be less natural and traditional than urban residents; they also perceive other oil-based materials to be less lasting, more timeless and, what is surprising, slightly more eco-friendly compared to urban residents.

Finally, we analysed the perception of the attributes of each material according to the age categories of our respondents. Figure 4c shows that the trend of responses for the attributes of wood is very similar in all age categories, with lower values assigned to all of the attributes by young respondents. The naturality and traditionality of wood are most appreciated by old respondents. The results for other natural materials are very similar, with similar trends of responses for each of the attributes. Nonetheless, old respondents assign the highest values to naturality and excitement compared to those assigned by respondents in the other two age categories.

In contrast, glass is perceived differently in the three age categories. Old respondents assign markedly higher values to traditionality compared to the other two age categories. Middle-aged respondents assign lower values to modernity, lastingness, and naturality compared to old respondents. Young respondents assign considerably lower values to naturality, excitement, and eco-friendliness compared to respondents in the other two age categories.

For metal, paper, and bio-based materials, the trends are very similar across all ages, with lowest assigned values for every attribute assigned by young respondents. The same trend of responses in all age categories is also found for plastic and other oil-based materials. However, for these materials, the lowest values are assigned by middle-aged respondents.

## Discussion

Understanding and engaging the public is key for ensuring the success of government and industry initiatives aimed at addressing the problem of the transition to the bioeconomy (Laibach et al. 2019; Lynch et al. 2016; Sijtsema et al. 2016). The acceptance increases when people feel more engaged with a technology or material, or when they expect a personal benefit through that technology or material (Lynch et al. 2016).

Results from other studies (Dilkes-Hoffman et al. 2019a; Lynch et al. 2016; Sijtsema et al. 2016) show that participants generally favour bio‐based technologies as a contribution to economic growth and sustainability, even though they are not familiar with bio-based technologies and materials. Surprisingly, bio-based materials are not as popular among the Slovak public as natural materials, even though both groups of materials come from living matter. The eco-friendliness of bio-based materials is not strongly perceived either, which means that respondents are reluctant towards the diversified and modern use of natural materials. These results contradict findings from the BIOWAYS project, which was carried out in 2016 and focused on analysing the public perception of bio-based products in several European countries, including Slovakia. According to the results of the BIOWAYS project, 80% of respondents perceived bio-based products positively. On the contrary, in our case, the majority of respondents has a neutral attitude towards bio-based materials. Although the Slovak public perceives bio-based materials in a more or less neutral way, these materials are still preferred over plastic and oil-based materials, which are in agreement with several studies (Magnier and Schoormans 2015, 2017). The preference for natural and bio-based materials might arise from the public perception of these materials as being environmentally friendly, helping to reduce air and soil pollution, and health risks (Magnier and Crié 2015), but also as being traditional, reliable and lasting materials of high quality. The neutral perception of bio-based materials by the Slovak public could originate from a lack of knowledge and information about these materials, as pointed out also in the Open-Bio project (Meeusen et at. 2015). Spierling et al. (2018) observed that the lack of knowledge might depend on the little usage of these materials. Poor awareness can cause mixed positive and negative associations that can heavily affect the perception.

Our results indicate the leading position of wood and other renewable materials in the Slovak market, which is mostly based on their naturality, traditionality, and eco-friendliness. Wood is generally preferred because it is perceived as natural and unprocessed. The properties of wood that are appreciated are connected to its natural origin. Wood-specific properties are highly valued as well, as they combine harmony and activity without disturbing irregularities (Jonsson et al. 2008; Paluš et al. 2012). Another wood product that is well perceived is biomass (Scarlat et al. 2015). This is relevant as trends show that the use of forest biomass will be continually increasing until 2030 (Hurmekoski et al. 2019).

In a Thai study concerning packaging materials, respondents preferred non-toxic packaging materials for prolonging the quality of product (Silayoi and Speece 2004). This agrees with our results, as the Slovak public prefers paper and glass over plastic and other oil-based materials. Dilkes-Hoffman et al. (2019a) also confirmed that the public prefers paper and glass compared to conventional plastic, yet plastic was not rated significantly different from biodegradable plastics. Plastics are viewed as a serious environmental issue and have been associated with food packaging, convenience, and environmental concern.

According to our results, women appear to have higher environmental awareness than men. According to Simon (2010), women tend to perceive biotechnology in general less favourably than men. This contradicts our results, according to which bio-based materials, that are dependent on biotechnology, are perceived in the same way by men and women. Women perceive natural materials and glass more positively, and oil-based materials more negatively than men, which could be caused by a higher knowledge of environmental sciences, as pointed out by Mohai (1991), and by the fact that women do not tend to place as high importance on economic costs (Caricati 2007). According to several studies, women are also more willing to reduce, reuse, and recycle products and materials (Kurisu and Bortoleto 2011), and to use alternative products to oil-based products, e.g. plastic bags (Madigele et al. 2017). A large number of studies found little or no relationship between demographic characteristics and environmental attitudes and behaviour, as demographic variables have less explanatory power than psychographic variables (Schwepker and Cornwell 1991). Thus, results from gender-based investigations are still far from being conclusive (Chen and Chai 2010), and warrant further research.

Rural residents perceive natural materials more positively compared to oil-based materials. This, we believe, is due to the greater connection with nature in rural areas. It also indicates naturally higher environmental awareness in comparison with urban residents. This is supported by the results of our analyses: old respondents tend to perceive natural materials more positively than middle-aged respondents, and significantly more positively then young respondents. We assume this to be in line with the European trend of young people preferring to live in or nearby large cities, while older people choose to live in smaller towns or in rural areas (Eurostat 2016,3). On the other hand, it was established by a French study that younger respondents are more willing to accept a reduction in their comfort to reduce the negative impact of human civilisation to the environment (Elgaaïed-Gambier 2016). This is slightly in contradiction with our results: even though, in our case, the young generation prefers natural materials, this preference is not as strong as that shown by middle-aged and old respondents.

A main characteristic of bioeconomy is its interdisciplinarity, as it involves different stakeholders from diverse economic sectors and regions, each with their own perspective (Laibach et al. 2019). Our study focused only on consumers. As discussed above, people perceive bio-based materials positively, even though they do not have enough knowledge about them. In order to make preferences, they seek for reliable information on both the advantages and disadvantages of bio-based materials to be able to make their own judgement (Sijtsema et al. 2016). Consumers and general public are more society-conscious than experts or other stakeholders, as they are not influenced by their area of expertise. While studying experts´ view on bioeconomy, social sustainability concerns were not taken into account as a criterion for bioeconomy development (Laibach et al. 2019). Bioeconomy experts around the world prioritise fields and criteria depending on their professional background and origin (Laibach et al. 2019). Some authors argue that the bioeconomy is very technology-driven and should be focused on high-end technologies and materials (e.g. Popescu 2014). Laibach et al. (2019) make the argument that the most important path to successfully implementing and enlarging the bio-based aspect in our current economy is the improvement of agriculture and the more efficient utilisation, including cascade and circular concepts, of biomass.

While policies often claim that the bioeconomy is part of the solution to environmental concerns (Schmid et al. 2012; Staffas et al. 2013), literature argues that without care it will cause even more problems (Philp 2015). Social sustainability concerns did not often appear as criteria suggestions in the bioeconomy development among experts (Laibach et al. 2019). While in the concept of a green economy, social concerns are implemented, political bioeconomy strategies are more focused on economic growth and job creation (Scarlat et al. 2015; UNEP 2011). While bioeconomy is considered to produce many opportunities, there are studies pointing at the gap between bioeconomy and sustainable development goals (SDG) caused mainly by oversimplification of bioeconomy conceptualization (Siegner et al. 2017). Incautious bioeconomy transformation may cause the rise of conflicts between some SDG, such as conflicting needs for food and bioenergy production, which leads to biodiversity loss, land use change, deforestation, etc. (Laibach et al. 2019).

Policy papers typically represent condensed strategic discourses and action programmes that are based on compromises between institutions (ministries and agencies) and different elites (business, politics, some academia), and which do not necessarily reflect the positions, worldviews, and opinions of a wide range of other stakeholders (Hausknost et al. 2017). Therefore, we can see a considerable gap between policy papers and visions supported by various stakeholders, including scientists, professionals, and the public.

This study as any other research is subject to some limitations that can serve as starting points for further discussion or research. One aspect is that the analysed materials and statements about their attributes were not selected by the authors. We were able to use data obtained from a survey conducted from the perspective of bio-based materials producers’ point of interest. Another limitation concerns the sample, as the age group over 61 was underrepresented due to the character of the survey (administered via the internet). It is therefore recommended for the future to combine data collection methods. Third, the direct linkage between the results and the potential acceptance of the bioeconomy cannot be clearly drawn, but can serve as an important impulse for further discussion. Finally, the results presented in this paper are limited to Slovakia. Further research would compare the results in other countries where the survey was run. However, we speculate that our findings may be extrapolated to countries with similar socio-economic history and natural conditions as Slovakia in Central and Eastern Europe. Certainly, this hypothesis could be tested in different geographical settings. Moreover, it could be explored whether aspects like environmental awareness, country origin, existence of bioeconomy strategy, etc., could influence the results. The efforts to raise awareness for the transition towards the bioeconomy, not only in the EU but also in the rest of the world, may provide a fruitful environment for further research on stakeholder and customer preferences.

The findings of this study are relevant for policy makers as they reveal public perceptions about renewable and non-renewable materials. If a bioeconomy strategy is going to be adopted in Slovakia, changes in the information policy instruments will have to be made to raise the awareness of Slovak citizens towards other modern and sophisticated renewable materials besides wood, glass, and paper, as well as their properties, specifically in the young and urban generation which constitutes the bulk of future buyers. As shown by other research, people predominantly place the responsibility for information provision on industry and government (Lynch et al. 2016; Sijtsema et al. 2016). To overcome the manifold challenges connected to the rise of the bioeconomy, such as public acceptance or regulatory restrictions, the demand of investments in R&D as well as policy investments have to increase. This is necessary also to make the bioeconomy more competitive compared to fossil-based products while staying in accordance with SDG especially in terms of social and environmental sustainability (Laibach et al. 2019).

## Conclusions

Slovak consumers prefer natural renewable materials over non-renewable materials, confirming our initial hypothesis, even when it comes to their lastingness, which is a well-known advantage of non-renewable materials, such as plastic. The preference for the renewable materials in general was also confirmed by the analysis of individual material attributes. This also implies that the public opinion in Slovakia is open to the societal transition towards bioeconomy and could support such efforts regardless of gender, age, or place of residence. Increased public awareness about bio-based materials and dissatisfaction about current trends in non-renewable material use is promising for bioeconomy development. Although our respondents perceive natural and bio-based materials more positively compared to oil-based materials, whether they would actually prefer these materials as consumers is debatable. Therefore, there is a window of opportunity for further research aimed at whether and to what extent public perception is reflected in consumer behaviour in Slovakia.

We see this work as a primary baseline for analysing the potential of bioeconomy in Slovakia through the public perception of different kinds of materials. Based on this analysis, we can improve the targeting for bioeconomy promotion in Slovakia, which can be helpful for developing the national bioeconomy strategy. In Slovakia, there is an urgent need for developing this strategy, but for the societal transition to be successful and unentangled, further societal and technological research is needed. Our results can likely be utilised in countries with similar socio-economic histories and, to a certain extent, natural conditions. They can provide a valuable input to the discussion on the development of national bioeconomy strategies.

## Footnotes

1 https://ec.europa.eu/eurostat/statisticsexplained/index.php?title=Europe_2020_indicators__climate_change_and_energy#General_overview.

2 https://ec.europa.eu/eurostat/statisticsexplained/index.php?title=File:Share_of_renewable_energy_in_gross_final_energy_consumption,_by_sector,_EU-28,_2000-2016_(%25).png.

3 https://ec.europa.eu/eurostat/statisticsexplained/index.php/Urban_Europe_%E2%80%94_statistics_on_cities,_towns_and_suburbs.
